# Pharmacist-physician collaborative care for outpatients with left ventricular assist devices using a cloud-based home medical management information-sharing system: a case report

**DOI:** 10.1186/s40780-020-00188-2

**Published:** 2021-02-01

**Authors:** Yoshiki Katada, Atsushi Yonezawa, Momoe Utsumi, Noriaki Kitada, Yu-ki Sato, Katsuyuki Matsumura, Asami Sukeishi, Shunsaku Nakagawa, Satoshi Imai, Takayuki Nakagawa, Kenji Minakata, Hideo Kanemitsu, Kenji Minatoya, Shinichi Nomoto, Kazuo Matsubara

**Affiliations:** 1 Department of Clinical Pharmacology and Therapeutics, Kyoto University Hospital, 54 Kawahara-cho, Shogoin, Sakyo-ku, Kyoto, 606-8507 Japan; 2 Department of Health Sciences, Graduate School of Medicine, Osaka University, 2-2 Yamadaoka, Suita, 565-0871 Japan; 3 Department of Cardiovascular Surgery, Graduate School of Medicine, Kyoto University, 54 Kawahara-cho, Shogoin, Sakyo-ku, Kyoto, 606-8507 Japan; 4 Department of Human Health Science, Graduate School of Medicine, Kyoto University, 53 Kawahara-cho, Shogoin, Sakyo-ku, Kyoto, 606-8507 Japan

**Keywords:** Warfarin, Anticoagulation, Pharmacist, Prothrombin time-international normalized ratio, Self-testing, Left ventricular assist device, CoaguCheck® XS, Cloud-based home management system

## Abstract

**Background:**

The standard anticoagulation therapy for patients implanted with left ventricular assist devices (LVADs) includes warfarin therapy. We developed a cloud-based home medical management information-sharing system named as LVAD@home. The LVAD@home system is an application designed to be used on iPad tablet computers. This system enables the sharing of daily information between a patient and care providers in real time. In this study, we reported cases of outpatients with LVADs using this system to manage anticoagulation therapy.

**Case presentation:**

The patient, a man in his 40s with end-stage heart failure owing to non-ischemic dilated cardiomyopathy, underwent LVAD implantation and warfarin was started on postoperative day 1. He started to use LVAD@home to manage warfarin therapy after discharge (postoperative day 47). He sent his data to care providers daily. By using this system, the pharmacist observed his signs of reduced dietary intake 179 days after discharge, and after consulting the physician, told the patient to change the timing of the next measurement earlier than usual. On the next day, the prothrombin time-international normalized ratio increased from 2.0 to 3.0, and thus the dose was decreased by 0.5 mg. Four patients used this system to monitor warfarin therapy from October 2015 to March 2018. In these patients, the time in therapeutic range was 90.1 ± 1.3, which was higher than that observed in previous studies. Additionally, there were no thromboembolic events or bleeding events.

**Conclusions:**

The cloud-based home management system can be applied to share real-time patient information of factors, including dietary intake that interact with warfarin. It can help to improve long-term anticoagulation outcomes in patients implanted with LVAD.

## Background

Left ventricular assist devices (LVADs) were developed to bridge a patient to heart transplantation [1]. However, as the blood of a patient with an LVAD is exposed to a large surface area of artificial materials, the patient is at a high risk of thrombus formation and thromboembolism. The standard anticoagulation therapy for patients implanted with an LVAD includes warfarin in addition to aspirin therapy [2]. Anticoagulation therapy with warfarin requires precise dose adjustments based on the periodic monitoring of prothrombin time-international normalized ratio (PT-INR) to minimize the risk of bleeding. Many factors, such as drug-drug interactions and lifestyle, can affect a patient’s response to warfarin [3, 4]. Sufficient monitoring of these factors is important for the management of warfarin therapy in outpatients. Recently, the development of accurate, portable, point-of-care PT-INR monitors has made possible patient self-testing (PST) as a feasible and convenient anticoagulation management strategy. Furthermore, point-of-care devices, including the CoaguCheck® XS (Roche Diagnostics, Mannheim, Germany) allow PT-INR testing at home [5, 6]. PT-INR PST improves the overall prognosis of patients on lifelong oral anticoagulation therapy because patients remain more precisely within the target therapeutic PT-INR range [7]. However, conventional warfarin therapy in outpatients does not facilitate the sharing of information other than the PT-INR and warfarin dose between patients and care providers in real time.

**Fig. 1:**
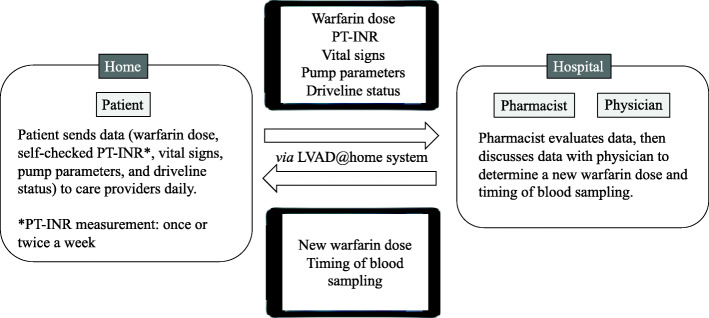
The outline of warfarin therapy using cloud-based home management system

We developed a cloud-based home medical management information-sharing system named as LVAD@home [8]. The LVAD@home system, an application designed to be used on iPad tablet computers, enables data on multiple factors, such as warfarin dose, self-checked PT-INR, blood pressure, dietary intake, body condition, body temperature, body weight, urine color, and other physical conditions, to be recorded and shared daily between patients and care providers in real time. Figure 1 shows the outline using this cloud-based home management system to manage warfarin therapy. However, it has been unclear whether this system is effective in the management of anticoagulation therapy. In this study, we reported cases of outpatients with LVADs using this cloud-based home management system to manage anticoagulation therapy.

## Case presentation

Patient No. 1 was a 40_S_-year-old male with end-stage heart failure owing to a non-ischemic dilated cardiomyopathy underwent an implantation with Heart-Mate II® LVAD (Abbott, IL, USA) and anticoagulation therapy with warfarin was started postoperative Day 1. The clinical characteristics and outcomes of him are shown in Tables 1 and 2. The target range for PT-INR was set at between 1.8 and 2.6. Before discharge, he attended an education/training program conducted by ward pharmacists for approximately 60 min. In this program, the pharmacist taught important aspects of oral anticoagulation therapy (importance of adherence, target PT-INR ranges, how to use CoaguCheck® XS, signs and symptoms of bleeding and clotting and appropriate actions in the event of these problems, drug interactions, avoiding vitamin K-rich food, and lifestyle issues including dietary intake). If the patient forgot to send data to care providers, the care providers have asked the patients to enter data via LVAD@home system or by telephone. In case of emergency event such as bleeding and pump trouble, the patient must contact hospital by telephone, not LVAD@home system. After discharge (postoperative Day 47), the patient started to use the LVAD@home system to share clinical information with care providers daily. PT-INR was assessed using a CoaguCheck® XS once or twice a week. The pharmacist could evaluate the data based on a treatment algorithm of warfarin developed with physicians [11]. In the case of abnormalities, the pharmacist discussed the data with the physician to determine a new warfarin dose and sent the new dose and timing of blood sampling via this system. After hospital discharge, the patient had good control of PT-INR without adverse events, such as bleeding.

**Table 1:** Baseline clinical characteristics of patients with an LVAD

No	1	2	3	4
Gender	Male	Male	Male	Male
Age (years)	40s	10s	50s	40s
LVAD device	HM II	HM II	HM II	HM II
Preimplant device strategy	BTT	BTT	BTT	BTT
Period of LVAD@home system (days)	581	643	464	400
PT-INR results of CoaguCheck® XS (times)	53	77	58	51
Co-administration of any antiplatelet agent	Aspirin	Aspirin	Aspirin	Aspirin
Target PT-INR	1.8–2.6	1.8–2.6	1.8–2.6	1.8–2.6 →2.5–3.5

*LVAD* Left ventricular assist device, *PT-INR* Prothrombin time-international normalized ratio, *HM II* Heart-Mate II, *BTT* Bridge-to-transplant

**Table 2:** Clinical outcomes of patients with an LVAD

No	1	2	3	4	Bishop et al. [9]	Ryan et al. [10]
TTR (%)	88.6	92.2	89.6	90.1	44.4	74.0
Overall PT-INR, mean ± SD	2.01 ± 0.30	1.87 ± 0.21	1.92 ± 0.22	2.47 ± 0.50	2.32 ± 0.67	–
Overall warfarin dose, mean ± SD	4.49 ± 0.76	5.13 ± 0.58	3.30 ± 0.34	3.24 ± 0.33	–	–
Major bleeding events						
GI bleeding events	no	no	no	no	yes (2/11)	no
LVAD site bleeding events	no	no	no	no	no	no
Other bleeding events	no	no	no	no	no	no
All thrombosis events						
Pump thrombosis events	no	no	no	no	no	no
Other thrombosis events	no	no	no	no	yes^a^ (1/11)	yes^b^ (2/162)
LVAD-related infections	no	yes	yes	no	–	–
Heart transplants received	no	yes	no	no	yes (1/11)	–

*TTR* Time in therapeutic range, *PT-INR* Prothrombin time-international normalized ratio, *GI* Gastrointestinal, *LVAD* Left ventricular assist devices

^a^Central nervous system thrombosis, ^b^Deep vein thrombosis

**Fig. 2:**
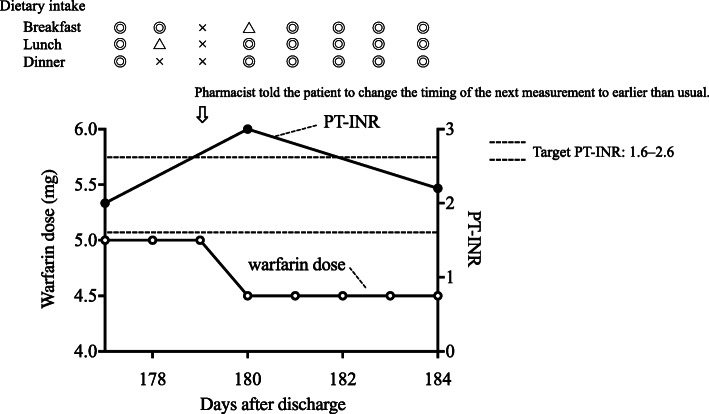
Time course of warfarin therapy using cloud-based home management system in patient No. 1. Closed and open circles show the PT-INR and warfarin dose, respectively. *X*-axis represents the days after discharge. Arrows indicate when the pharmacist told the patient to change the timing of the next measurement to earlier than usual

On Day 179 after discharge, the pharmacist observed signs of reduced dietary intake on the LVAD@home system. The reason of reduced dietary intake was that the patient was too busy with work. The pharmacist, after consulting the physician, told him to change the timing of the next measurement to earlier than usual (Fig. 2). In addition, the pharmacist explained about the influence of dietary intake on an efficacy of warfarin. On the next day, the patient measured his PT-INR, and found its prolongation from 2.0 to 3.0. Based on this information, the physician decided to decrease the dose of warfarin by 0.5 mg. The physician entered the message to reduce the dose of warfarin from 5.0 mg to 4.5 mg via LVAD@home. After taking warfarin, the patient input warfarin dose. The care providers could know that they took appropriate warfarin dose. Thereafter, his dietary intake returned to the baseline and he maintained a stable PT-INR value.

Four patients were using this system to monitor warfarin therapy from October 2015 to March 2018. The demographics and clinical characteristics of this study population are shown in Table 1. The mean age of all patients was 41 ± 14 years (mean ± SD) and all were male. All patients underwent LVAD placement as bridge-to-heart transplant therapy. They were administered warfarin and aspirin after the LVAD implantation. The target PT-INR level of patients No. 1–3 was set between 1.8 and 2.6 by the cardiac surgeon. For patient No. 4, the goal for PT-INR was increased from 1.8–2.6 to 2.5–3.5 because of the rising lactate dehydrogenase levels. The mean duration of warfarin therapy in all outpatients was 522 ± 95.3 days. The mean number of home PT-INR measurements by CoaguCheck® XS was 59.7 ± 10.2.

The clinical outcomes of the four patients are described in Table 2. The quality of anticoagulation management is expressed as the duration ratio in the target therapeutic range of PT-INR (time in therapeutic range [TTR]) [12]. The mean TTR of all patients was 90.1 ± 1.3. There were no thromboembolic and bleeding events. Furthermore, patient No. 2 underwent a successful heart transplantation after 643 days from starting usage of this system.

## Discussion and conclusions

Telephone and e-mail are usually used to share information on home-based PST, such as warfarin dose and PT-INR, between a patient and care providers to manage warfarin therapy [9, 13, 14]. However, with these tools, information on vital signs cannot be shared in real time. A change in vital signs is an important indicator of the anticoagulative efficacy of warfarin; hence, the real-time sharing of this information provides opportunities for early recognition and intervention. Physicians and pharmacists need to access to the health information of a patient in real time to decide on a warfarin dose in clinical practice. Recently, we reported that the anticoagulation activity of warfarin is significantly associated with fasting [15]. When patients experience fasting, more frequent PT-INR measurements and subsequent dose adjustments of warfarin are warranted to avoid adverse effects, such as bleeding. In this study, inadequate anticoagulation (PT-INR was prolonged from 2.0 to 3.0) was observed in one patient due to a decreased dietary intake. Daily follow-up is mandatory, even in the context of home management, and the early detection of reduced dietary intake is important to improve anticoagulation therapy. Using this cloud-based home management system, care providers can remotely track the health information of a patient, such as self-checked PT-INR, warfarin dose, and vital signs, in real time. This system strengthens the communication between the patient and care providers.

The TTR, a surrogate marker for the quality of anticoagulation control, is used to evaluate the clinical effectiveness and safety of warfarin [16]. Significant reduced risk of stroke have been reported in patients with atrial fibrillation taking warfarin whose TTRs are > 70% compared with those not receiving warfarin [17]. Previous studies have reported that anticoagulation management using PST achieves better control of PT-INR and reduces the risk of bleeding and thrombotic events in patients with atrial fibrillation, venous thromboembolism, and LVADs [9, 10, 18–20]. In this study, using LVAD@home system monitoring, anticoagulation therapy with warfarin was well-controlled in four patients, as their TTR was more than 90% without thromboembolic and bleeding events. Furthermore, the mean TTR in our study was higher than that in previous studies [9, 10]. Therefore, the LVAD@home system may improve long-term anticoagulation outcomes in patients implanted with LVAD with PT-INR self-testing at home.

In conclusion, the cloud-based home management system can be applied to share real-time patient information of factors, including dietary intake, that interact with warfarin. It can improve long-term anticoagulation outcomes in patients implanted with LVAD.

## Data Availability

Data used in this case report will not be shared owing to the risk of identifying an individual.

## Abbreviations

LVAD: Left ventricular assist devices
PT-INR: Prothrombin time-international normalized ratio
PST: Portable patient self-testing
TTR: Time in therapeutic range
